# Account of Diseases in an Eastern District of London, from the 20th of April, to the 20th of May, 1801

**Published:** 1801-06

**Authors:** 


*Account of Diſeaſes in an Eaſtern Diſtrict of London, from the 20th of April, to the 20th of May,* 1801.

| | No. of Caſes. | | No. of Caſes. |
|---|---|---|---|
| ACUTE DISEASES. | | Cynanche Tonſillarum | 2 |
| Typhus | 14 | Ophthalmia | 2 |
| Pneumonia | 7 | Apoplexia | 2 |

CHRONIC

| CHRONIC DISEASES. | |
|---|---|
| Cough - - - - - - - | 25 |
| Dyfpnœa - - - - - - | 15 |
| Phthifis Pulmonalis - - | 5 |
| Hydrothorax - - - - - | 3 |
| Anafarca - - - - - - | 5 |
| Cephalalgia - - - - - | 7 |
| Vertigo - - - - - | 2 |
| Hepatitis Chronica - - | 2 |
| Enterodynia - - - - - - | 4 |
| Diarrhœa - - - - - - | 15 |
| Dyfuria - - - - - - - | 2 |
| Amenorrhœa - - - - - | 7 |
| Fluor Albus - - - - - | 4 |
| Chlorofis - - - - - - - | 3 |
| Dyfpepfia - - - - - - | 6 |
| Vomitus - - - - - - | 3 |
| Herpes Puftulofus - - - - | 4 |
| Lumbago - - - - - - | 2 |
| Rheumatifmus Chronicus - | 15 |
| PUERPERAL DISEASES. | |
| Mania - - - - - - - | 1 |
| Menorrhagia Lochialis - | 3 |
| Lactea - - - - - - - | 2 |
| Maftodynia - - - - - | 3 |
| INFANTILE DISEASES. | |
| Hydrocephalus Internus - | 1 |
| Vermes - - - - - - - - | 2 |
| Febris - - - - - - - - | 2 |
| Diarrhœa - - - - - - | 3 |
| Herpes Miliaris - - - - | 2 |

The fever which has fo long raged, and which has been attended with various affections of the head, has for a few weeks appeared lefs frequently. This difeafe, however, has not entirely fubfided: after a fhort fufpenfion it has returned, but with lefs aggravation of fymptoms, and the number of patients affected by it has been confiderably lefs than formerly. Amongft thofe cafes which have been under the care of the Reporter, there have been fome, the fymptoms of which have differed from thofe which occurred in former inftances. Thofe affections of the head, which in them formed fo characteriftic a feature, have been lefs frequent in their occurrence, and lefs violent in their effects. In fome inftances, affections of the refpiratory organs have formed a more ftriking fymptom than thofe of the head, and have rendered neceffary the application of leeches, and of blifters to the cheft.

The influence which the paffions of the mind have on the different functions of the fyftem, has long formed a fubject of curious inveftigation by the Metaphyfician and Pathologift. The debilitating effects of fudden fear, or of long protracted grief, and the more immediately deftructive influence of violent anger or rage, have been exemplified in too many painful inftances; whilft the more pleafing effects of hope and joy have been felt in the general ftate of health and fpirits. The latter of thefe paffions, however, when *fuddenly* raifed, or indulged to excefs, has fometimes been attended with fatal confequences. In one of the inftances of apoplexy referred to in the lift of difeafes, the patient experienced a furprize of joy, at reading in the newfpaper of the arrival of a fhip, in which a near relation was a paffenger, concerning whofe fafety he had before expeexperienced confiderable anxiety. He immediately related the fact to a friend with great emotion, fell upon the floor with the paper in his hand, and in a few hours expired.

---

### *Prof. Hufeland on the Erysipelas of new-born Children.*

